# Fellow-Workers and the Roll of Honour

**Published:** 1915-08-14

**Authors:** 


### ROUND THE WORLD.

**[The Editor invites Early Information and Contributions Marked "Fellow-Workers."]**

### Fellow-Workers and the Roll of Honour.

Lieutenant Geoffrey Montague Mason Fleming, R.A.M.C., whose name recently appeared amongst those killed in Flanders, was an M.B., B.Ch. of Dublin. Qualifying in 1913, he joined the Army in the very first days of the war.

Lieutenant-Colonels A. Balfour, G. S. Buchanan, and L. Dudgeon, who are working with Colonel William Hunter (Charing Cross Hospital) in the R.A.M.C., with special reference to the control of epidemic disease amongst the Mediterranean troops, form a remarkably strong combination of experts on public health, hæmatology, and pathology.

Lieutenant-Colonel Andrew Balfour, R.A.M.C., C.M.G., is the well-known director of the Wellcome Bureau of Scientific Research, and was at one time medical officer of health at Khartoum, where he carried out very important work in the extermination of insect-borne diseases. An Edinburgh man, he qualified in 1892, and subsequently became a B.Sc., D.P.H., and M.D., taking a gold medal at the examinations for the last-named degree. He is also an F.R.C.P. Edin., and has written extensively on difficult problems of preventive medicine, particularly in regard to tropical countries.

Lieutenant-Colonel George Seaton Buchanan, R.A.M.C., occupies an important position as first assistant medical officer to the Local Government Board, under which he was formerly a medical inspector and chief inspector of foods. A student of University College and St. Bartholomew's Hospital he qualified in 1891, winning the gold medal in forensic medicine at the M.B. Lond. Previously to this Dr. Buchanan had taken the B.Sc. Lond., and he completed the London University course by becoming an M.D. in 1892. He has reported to the Local Government Board on numerous problems of importance in regard to public health.

The brilliant work of Colonel William Hunter and Lieutenant-Colonel Leonard Dudgeon, at Charing Cross and St. Thomas's Hospitals respectively, has previously been commented on in these notes.

In the recent report of the Imperial Research Fund it is interesting to note that four members of the staff of investigators are now on active service with the R.A.M.C., their special knowledge and technical skill being, of course, extremely useful in the elucidation of various bacteriological and sanitary problems affecting the health of the troops. These investigators are Dr. William Ewart Bullock, M.D., gold medallist at Edinburgh two years ago; Dr. Arthur Compton, an authority on physiological and pathological chemistry; Dr. Bertie Ronald Gordon Russell, a former Anderson scholar and McRobert Fellow of Aberdeen University; and Dr. Charles Singer, formerly research registrar at the Cancer Hospital and physician to out-patients at the Seamen's (*Dreadnought*) Hospital, Greenwich.

The *St. Thomas's Hospital Gazette* announces that the following St. Thomas's men have lately joined the R.A.M.C. as temporary lieutenants: W. H. Allen, R. A. Banbury, C. E. Bashall, E. C. Bourdas, H. J. de Brent, G. W. FitzHenry, W. Gilbertson, J. D. Gimlette, S. H. Hall, W. Haward, W. D. Knocker, M. H. Montesole, V. C. Pennell, M. J. Petty, F. C. Pridham, H. R. Sedgwick, H. B. Shepherd, F. W. S. Stone, W. J. F. Symons, A. G. J. Thompson, G. W. Thompson, H. C. Thorp, J. N. Wheeler, and A. P. Yonge.

Amongst medical casualties reported from the Near East is the death of Captain Michael Foster Reaney, I.M.S., killed in action. Qualifying from the London Hospital in 1900, he became house physician there and also held various non-resident posts in the out-patient department, after which he obtained a commission in the I.M.S. in the early part of 1905.

Captain Andrew Wallace, recently killed, was a medical man who lost his life as a combatant officer. Qualifying by taking the M.B., C.M. in 1896, he was for a time in private practice, and then served as ship's surgeon with the "Glen" steamers, ultimately settling down as a medical practitioner in Berwickshire. A keen "Volunteer," Captain Wallace had been attached to his regiment, the 4th King's Own Scottish Borderers, for twelve years or more, and had ranked as captain for some little time.

The steadily increasing list of medical students who have died on the field of battle now includes the names of Mr. Andrew Hubert Millin Henderson and Mr. J. S. Stewart. The first-named had finished about a year of the medical curriculum at Edinburgh when his call came, and he went to the Front as second-lieutenant in the 1/4th King's Own Scottish Borderers, having, as a matter of fact, obtained his commission in that regiment a year or so previously. He was the son of Dr. P. J. Henderson, of Galashiels. Mr. Stewart, who was the son of Dr. W. Stewart, of Gourock, was a second-lieutenant in the 5th Argyll and Sutherland Highlanders, having obtained his commission in the latter part of last year.

The staff of the St. John Ambulance Brigade Hospital at Etaples, near Boulogne, is to include four distinguished representatives of the Belfast Medical School—namely, Dr. T. Houston, Dr. J. E. MacIlwaine, Mr. P. T. Crymble, and Dr. McCloy.

Dr. Thomas Houston, M.D. (R.U.I.), occupies several important posts in Belfast, including those of physician in charge of the department of hæmatology and vaccine therapy at the Royal Victoria Hospital, pathologist to the Ulster Hospital, and joint lecturer in medical jurisprudence at Queen's College. Formerly he was assistant gynæcologist at the Royal Victoria Hospital, and a research scholar (1902-03) of the British Medical Association.

Dr. John Elder MacIlwaine, M.D. (R.U.I.), B.Sc. Glasg., D.P.H. Cantab., is assistant physician to the Royal Victoria Hospital, and visiting physician to the Forster Green Consumption Hospital. He served in South Africa as medical officer to the Irish Hospital.

Mr. Percival Templeton Crymble, M.B. (R.U.I.), F.R.C.S. Eng., is surgeon to out-patients at the Belfast Hospital for Sick Children, surgical registrar to the Royal Victoria Hospital, and lecturer in applied anatomy at Queen's College. His brother, Mr. C. R. Crymble, D.Sc., also a prominent Belfast graduate, went to the Front in the early days of the war as a lieutenant, and was killed in action.

